# Virus-Derived Small Interfering RNAs Affect the Accumulations of Viral and Host Transcripts in Maize

**DOI:** 10.3390/v10120664

**Published:** 2018-11-23

**Authors:** Zihao Xia, Zhenxing Zhao, Zhiyuan Jiao, Tengzhi Xu, Yuanhua Wu, Tao Zhou, Zaifeng Fan

**Affiliations:** 1 College of Plant Protection, Shenyang Agricultural University, Shenyang 110866, China; wuyh09@syau.edu.cn (Y.W.); 2 State Key Laboratory of Agro-Biotechnology and Key Laboratory of Pest Monitoring and Green Management-MOA, China Agricultural University, Beijing 100193, China; zhaozx@cau.edu.cn (Z.Z.); jiaozhiyuan.2009@163.com (Z.J.); xtz9012@gmail.com (T.X.); taozhoucau@cau.edu.cn (T.Z.)

**Keywords:** *Sugarcane mosaic virus* (SCMV), virus-derived small interfering RNA (vsiRNA), artificial microRNA (amiRNA), maize protoplast, degradome analysis

## Abstract

RNA silencing is a conserved surveillance mechanism against invading viruses in plants, which involves the production of virus-derived small interfering RNAs (vsiRNAs) that play essential roles in the silencing of viral RNAs and/or specific host transcripts. However, how vsiRNAs function to target viral and/or host transcripts is poorly studied, especially in maize (*Zea mays* L.). In this study, a degradome library constructed from *Sugarcane mosaic virus* (SCMV)-inoculated maize plants was analyzed to identify the cleavage sites in viral and host transcripts mainly produced by vsiRNAs. The results showed that 42 maize transcripts were possibly cleaved by vsiRNAs, among which several were involved in chloroplast functions and in biotic and abiotic stresses. In addition, more than 3000 cleavage sites possibly produced by vsiRNAs were identified in positive-strand RNAs of SCMV, while there were only four cleavage sites in the negative-strand RNAs. To determine the roles of vsiRNAs in targeting viral RNAs, six vsiRNAs were expressed in maize protoplast based on artificial microRNAs (amiRNAs), of which four could efficiently inhibit the accumulations of SCMV RNAs. These results provide new insights into the genetic manipulation of maize with resistance against virus infection by using amiRNA as a more predictable and useful approach.

## 1. Introduction

In plants, RNA silencing is a conserved mechanism against virus infections, which involves the production of virus-derived small interfering RNAs (vsiRNAs) [1,2]. These vsiRNAs are generated by DICER-like (DCL) proteins that recognize and process double-stranded RNA (dsRNA) from replication intermediates and highly structured single-stranded RNA (ssRNA), which guide the RNA-induced silencing complex (RISC) containing specific Argonaute (AGO) proteins to cleave viral RNAs and specific host mRNAs in a sequence-specific manner [3,4,5]. In virus-infected plants, the effect of RNA silencing can be amplified by secondary vsiRNAs that were derived from aberrant viral dsRNAs processed by RNA-dependent RNA polymerases (RDRs) [6]. As a response to RNA silencing defense, plant viruses encode viral suppressors of RNA silencing (VSRs), which can inhibit the function of RNA silencing machinery at multiple steps [7,8].

To understand the potential regulatory and biological functions, it is essential to find the target sites for si/miRNAs [9]. In plants, AGO-mediated cleavage of mRNA targets often takes place precisely between the 10th and 11th nucleotides from the 5′-end of miRNA, which is also valid for vsiRNA-mediated RNA cleavage [4]. The resulting 5′-uncapped and polyadenylated fragments of the cleaved target RNAs can be identified and characterized, although the upstream fragments are rapidly degraded [10]. To date, 5′-rapid amplification of cDNA ends (RACE) has been used to identify miRNA-mediated cleavage sites, yet it is only suitable on a small scale due to its low efficiency [9,11]. Recently, degradome analysis has been widely established in plants, which is a new and efficient strategy to identify si/miRNA-mediated cleavage sites on a large scale [11,12,13].

Artificial miRNA (amiRNA) is an artificial small RNA (sRNA) engineered to silence a specific transcript, which is produced by the expression of a native miRNA precursor with customized miRNA/miRNA* sequence [14]. Like miRNAs, amiRNAs derive from the DCL1 cleavage of miRNA precursors with stem-loop structures, of which the guide strand associates with an AGO protein to silence target transcripts usually by cleavage [15]. In plants, near-perfect complementarity between miRNA and its mRNA target is essential for silencing, which also ensures the high specificity of silencing [9]. Thus far, amiRNAs have been used to selectively silence plant endogenous genes [16,17], as well as viruses by modified amiRNA sequences for plant antiviral genetic engineering, which has been shown to confer virus resistance in several plant species [18,19,20]. Recently, transient expression systems in protoplasts have been described by overexpressing amiRNAs and target mimics to manipulate amiRNA levels and consequently impact on their targets [16,21,22], making it possible to monitor the efficacy of amiRNAs in silencing viral RNAs without transgenic plants.

*Sugarcane mosaic virus* (SCMV), belonging to the genus *Potyvirus* in the family *Potyviridae*, can infect various crops, such as maize (*Zea mays* L.), sugarcane (*Saccharum* spp) and sorghum (*Sorghum vulgare*), leads to typical symptoms including mosaic, chlorosis and dwarfing, and causes considerable losses in different field crops worldwide [23]. SCMV is the major causal agent of maize dwarf mosaic disease, and the Beijing isolate belongs to a prevalent strain in China [24,25]. Our previous studies have preliminarily characterized the vsiRNAs derived from SCMV by high-throughput sequencing and identified the target genes of maize miRNAs through degradome analysis in SCMV-infected maize plants [26,27]. However, how vsiRNAs function to target SCMV RNAs and/or maize transcripts has not been investigated. In this study, we further analyzed the datasets by integrating vsiRNAs and degradome library to identify the cleavage sites in SCMV RNAs and maize transcripts linked to vsiRNAs and amiRNAs were used to determine the roles of vsiRNAs. These results provided novel insights into the improvement of maize crop engineering against virus infection by making amiRNA a more predictable and useful technology.

## 2. Materials and Methods

### 2.1. Plant Growth and SCMV Inoculation

SCMV Beijing isolate (GenBank accession number AY042184) was from previously published sources [24]. Maize inbred line Zong31 was planted and inoculated with SCMV as described previously [27]. Systemic leaves were harvested at approximately 8 days post inoculation (dpi) for degradome sequencing, and at 4, 8 and 12 dpi for detecting the expression of vsiRNAs and their target genes, respectively. The collected samples were immediately frozen in liquid nitrogen and stored at −80 °C until use.

### 2.2. RNA Extraction, Degradome Sequencing and Bioinformatics Analysis

At least 15 maize seedlings inoculated with SCMV at 8 dpi were pooled for RNA extraction using TRIzol reagent (Invitrogen, Carlsbad, CA, USA). Degradome sequencing and analysis was performed as described previously [27]. CleaveLand 3.0 pipeline [28] and ACGT301-DGEv1.0 program (LC Sciences, Hangzhou, China) were used to identify and classify the categories of sliced vsiRNA target genes, including SCMV RNAs and maize transcripts. The categories of sliced target transcripts and the criteria of different categories were described in our previous report [27].

### 2.3. Plasmid Construction

All plasmids used in this work were listed in the Table S1. For pGD-GFP plasmid constitutively expressing green fluorescent protein (GFP) under 35S promoter, the full-length coding sequence was amplified by reverse transcription-polymerase chain reaction (RT-PCR), digested by *Xho*I/*Bam*HI (TaKaRa Bio Inc., Dalian, China) and inserted into the same digested pGD vector [29] for transient expression in maize protoplasts. For pGD-mGFP plasmid, a one-step overlapping PCR was firstly performed to make the GFP coding sequence mutant and constructed subsequently as pGD-GFP. The *Arabidopsis* miR319a precursor or rice miR528 precursor as the template was used to assemble the precursors for individual amiRNAs by a two-step overlapping PCR method as described previously [16]. PCR products of pre-amiRNAs were digested by *Sal*I/*Bam*HI (TaKaRa Bio Inc., Dalian, China) and inserted into the same digested pGD vector. For the plasmids expressing polycistronic amiRNAs, the second and the third pre-amiRNAs were obtained by digesting the first pre-amiRNAs by *Xba*I/*Bam*HI (TaKaRa Bio Inc., Dalian, China) and sequentially inserted into *Spe*I/*Bam*HI (TaKaRa Bio Inc., Dalian, China) digested sites downstream the first pre-amiRNA within the pGD vector.

### 2.4. Northern Blotting Analysis and Quantitative RT-PCR

Total RNA extracted as described above was used for detection of vsiRNAs by Northern blotting and reverse transcription after DNase I (TaKaRa Bio Inc., Dalian, China) treatment [26]. The procedure of Northern blotting and quantitative RT-PCR (qRT-PCR) were performed as reported previously [26]. The sequences of probes and primers used for Northern blotting and qRT-PCR were listed in Table S2 and Table S3, respectively.

### 2.5. Maize Protoplasts Transfection and Confocal Microscopy

Maize inbred line Zheng58 was used for protoplasts isolation and transfection as described previously [30]. Approximately 15 μg pGD-amiRNA and 5 μg pGD-GFP/mGFP or SCMV viral RNA from purified virus were gently mixed with 1 × 10^5^ maize protoplasts. The transfected protoplasts were harvested at 16 h post transfection and used for further analysis. Three independent experiments were conducted and at least 10 Eppendorf tubes of protoplasts were used for each treatment within each experiment, which were pooled for RNA isolation followed by qRT-PCR and Northern blotting analysis. For pGD-GFP/mGFP, another 10 tubes of protoplasts from the same treatment were pooled and used for protein extraction and Western blot assay as described previously [30].

The fluorescence signals of GFP and mGFP were visualized under a Nikon Eclipse TE 2000-E laser-scanning confocal microscope equipped with Nikon EZ-C1 FreeViewer 3.00 (Nikon, Tokyo, Japan) as described previously [31]. The fluorescence was excited at 488 nm. The images were captured at 16 h post transfection.

## 3. Results

### 3.1. Identification of Host Transcripts Targeted by vsiRNAs

**Figure 1:**
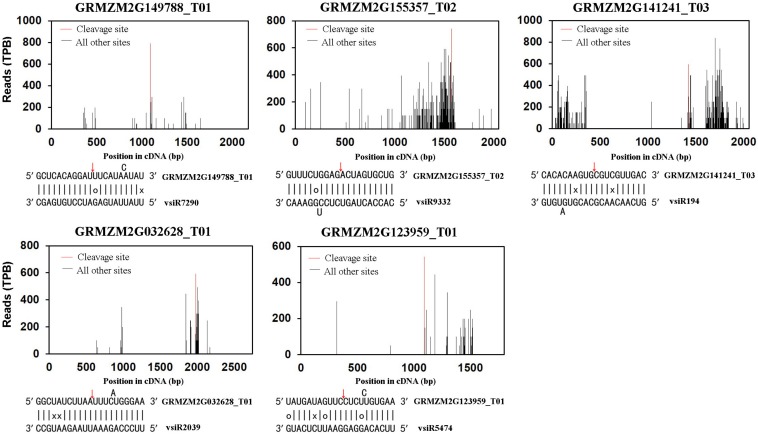
Identification of maize transcripts targeted by vsiRNAs through degradome sequencing. Target plots for the indicated genes showed sequencing signature abundance at the position of target transcripts identified through degradome sequencing. The red lines indicate the predicted vsiRNA cleavage sites. Signature abundance along the mRNA was normalized to the transcripts per billion (TPB) reads. The red arrows in the mRNAs represent the cleavage sites identified through degradome sequencing. Wobble G-U pairs are indicated with circles and no base pairing is indicated with a ‘×’.

Previously, we used a bioinformatics tool to integrate miRNAs from a small RNA library with a genome-wide map of uncapped transcripts (degradome) from the same SCMV-infected maize plants. Indeed, a number of maize transcripts targeted by conserved and novel maize miRNAs were identified [27]. In this study, we adopted the same method to identify maize mRNAs targeted by vsiRNAs from SCMV (Table S4). The results showed that 42 maize transcripts were targeted by vsiRNAs (Table S4), and the target plots of five transcripts with the most reads at the cleavage site were shown in Figure 1. These vsiRNAs possessed distinct size classes, among which 23 were 21 nucleotides in length, 12 were of 22 nucleotides and 7 were identified in both 21- and 22-nucleotide size classes. Moreover, all those vsiRNAs were of sense polarity, and the nucleotide at their 5′-ends was A in 15 cases, U in 12, G in 11 and C in four (Table S4).

Thirty-eight of the 42 targeted transcripts were annotated with putative functions in the maize genome and 14 were of category 0 (Table S4), indicating that the highest frequency of degradome tags mapped on the host transcripts in the expected positions of vsiRNA cleavage sites. vsiR2577, 2578 and 2581 could target the same transcript *GRMZM2G009443_T01* that encodes a putative chloroplastic chaperone protein ClpC1 [32]. Moreover, other targeted transcripts, including *GRMZM2G032628_T01*, *GRMZM5G806622_T01*, *GRMZM2G066234_T01*, *GRMZM2G130121_T01* and *GRMZM2G132847_T01*, also encode putative chloroplastic proteins (Table S4), which might be involved in chloroplast development and function [33].

**Figure 2:**
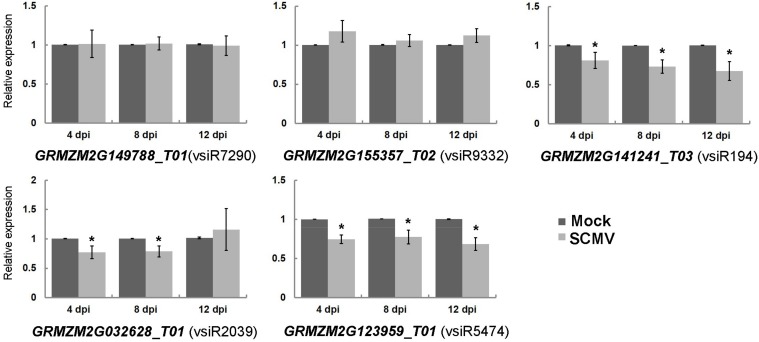
The expression of maize transcripts targeted by vsiRNAs in Mock- and SCMV-inoculated maize plants. The expression levels of the indicated target genes were determined by qRT-PCR at 4 dpi, 8 dpi and 12 dpi, respectively. Three independent experiments were conducted with at least three biological replicates each and the data were analyzed using a two-sample *t*-test. An asterisk indicates a significant difference (*p*-value < 0.05). Bars represent the means ± SD.

Endogenous small RNAs are known to down-regulate host transcripts mainly at the post-transcriptional level in plants [34]. Likewise, our analysis suggests that vsiRNAs from SCMV can promote the cleavage of the targeted transcripts in Table S4. To examine whether the accumulations of the transcripts were down-regulated upon SCMV infection, the qRT-PCR experiments were carried out to quantify the expressions of five targeted transcripts with the most reads at the cleavage site at three stages of SCMV infection (Figure 2). The results revealed that the accumulations of both *GRMZM2G141241_T03* and *GRMZM2G123959_T01* targeted by vsiR194 and vsiR5474 were decreased at all three stages of SCMV infection, respectively. However, SCMV infection has no significant effects on the accumulations of transcripts *GRMZM2G149788_T01* and *GRMZM2G155357_T02* targeted by vsiR7290 and vsiR9332 at all three stages, respectively. Interestingly, the transcript *GRMZM2G032628_T01* targeted by vsiR2039 was down-regulated at 4 and 8 dpi upon SCMV infection, while there was no significant change at 12 dpi. The discrete accumulations of targeted transcripts suggested the diversified regulation of these transcripts by vsiRNAs or other pathways after SCMV infection.

### 3.2. Analysis of Cleavage Sites on Viral Genomes Targeted by vsiRNAs

**Figure 3:**
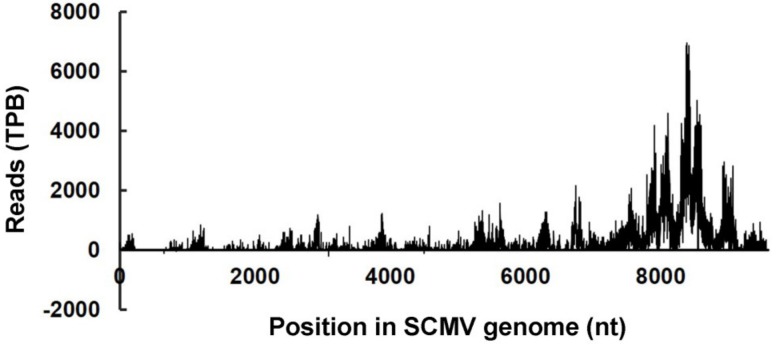
Distribution of vsiRNA-directed cleavage sites along the viral genome of SCMV. Reads of viral 5′-uncapped ends were normalized to the transcripts per billion (TPB) reads. Bars above the axis represent the cleavage sites in the sense strand of SCMV RNAs; those below represent the cleavage sites in the antisense strand of SCMV RNAs.

Our previous reports have demonstrated that large amounts of vsiRNAs accumulate in SCMV-infected maize plants [26]. Considering that vsiRNAs can be loaded into specific AGOs to degrade viral genomic RNAs by cleavage, we explored the cleavage sites on SCMV genomic RNAs targeted by vsiRNAs. SCMV, like other potyviruses, possesses a single-stranded, positive-sense RNA genome with M7G cap structure at its 5′-ends and a poly(A) tail at 3′-end [35]. These characteristics provided the possibility to identify cleavage sites on viral genomes by searching for virus-derived uncapped 5′-remnants within the degradome database (Figure 3 and Table S5).

The results revealed that a large amount of 5′-end specific uncapped sequences were from sense strand of SCMV genomes, while there were only four (656, 833, 3092, 4499) with low reads (99, 49, 198, 99) from the antisense strand. The uncapped sequences were almost continuously but heterogeneously distributed along the sense strand of the SCMV genome, and mainly concentrated in the 3′-proximal region. To identify the putative vsiRNA-mediated cleavage sites, we used the CleaveLand pipeline to search for vsiRNAs (both of 21 and 22 nucleotides) correlated with the datasets of SCMV degradomes. Almost all of cleavage sites could be explained by vsiRNAs, while the reads between cleavage sites and vsiRNAs were not consistent; despite the presence of many vsiRNA hotspots, only a few were associated with cleavage sites.

### 3.3. The Expression of miR-GFP Could Silence Its Target GFP in Maize Protoplast

**Figure 4:**
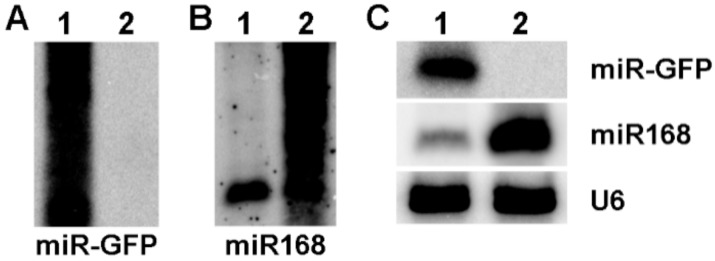
The expression of amiRNAs in maize protoplasts. (**A**) The expression of miR-GFP based on the precursor of ath-miR319a as backbone. (**B**) The expression of miR168 based on the precursor of ath-miR319a as backbone. (**C**) The expression of amiR-GFP and miR168 based on the precursor of osa-miR528a as backbone. U6 was used as a loading control. 1: The expression of miR-GFP; 2: The expression of miR168.

In this study, the precursor of ath-miR319a was used as the backbone of amiRNAs to express miR-GFP and miR168 in maize protoplasts as previously reported [16], respectively. The results of Northern blotting assays showed the existence of smear bands in both miR-GFP and miR168 expressed experiments (Figure 4A,B), suggesting that the process of the amiRNA precursor was not incomplete. Subsequently, the precursor of osa-miR528a was used and the clear bands of expressed miR-GFP and miR168 were shown (Figure 4C), indicating that the expression of amiRNAs based on the precursor of osa-miR528a was successful in maize protoplasts.

**Figure 5:**
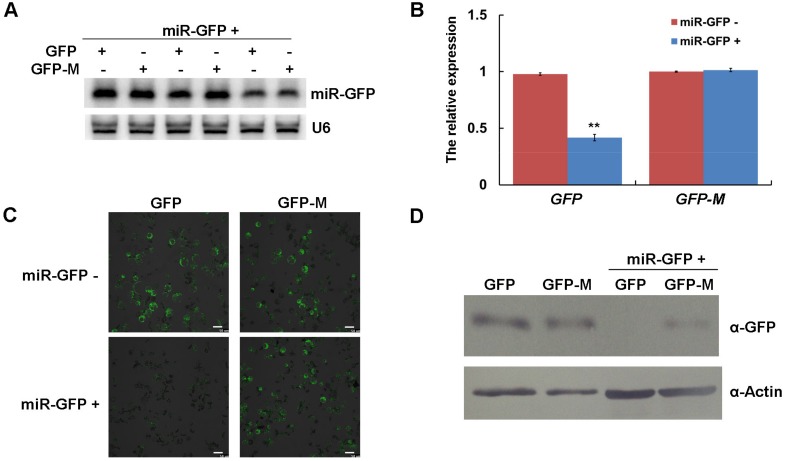
The expressed miR-GFP could silence the target GFP in maize protoplasts. (**A**) The expression of miR-GFP in maize protoplasts was detected by Northern blotting. Three independent experiments were conducted. U6 was used as a loading control. (**B**) The relative accumulation of *GFP* and *GFP-M* mRNAs in maize protoplasts was determined by qRT-PCR. Three independent experiments were conducted with at least three biological replicates and the data were analyzed using a two-sample *t*-test. Bars represent the grand means ± SD. “**” indicate a significant difference (*p*-value < 0.01). (**C**) The green fluorescence of GFP and GFP-M in maize protoplasts was observed with confocal microscopy, scale bar = 50 μm. (**D**) The expression of GFP and GFP-M in maize protoplasts was detected by Western blotting. Actin was used as a loading control.

To determine the suppression of the targets triggered by amiRNAs, we performed co-expression assays of miR-GFP and its target GFP in maize protoplasts. Northern blotting analysis confirmed the expression of miR-GFP in three independent experiments (Figure 5A). The results of qRT-PCR and Western blotting showed that the expressed miR-GFP could efficiently suppress the expression of its target GFP in both mRNA and protein levels (Figure 5B,D). However, the suppression was abolished when *GFP* carried a synonymously mutated version of the relevant miR-GFP target sites (Figure 5B,D). We also observed the weakened green fluorescence of GFP when co-expressed with miR-GFP at 16 h post transfection; when the miR-GFP target site of *GFP* was mutated, the brightness of green fluorescence was recovered (Figure 5C). These results of co-expression assays demonstrated that the expressed amiRNAs could efficiently suppress the expression of its target genes in maize protoplasts.

### 3.4. The Expression of vsiRNAs Could Silence SCMV RNA in Maize Protoplast

**Figure 6:**
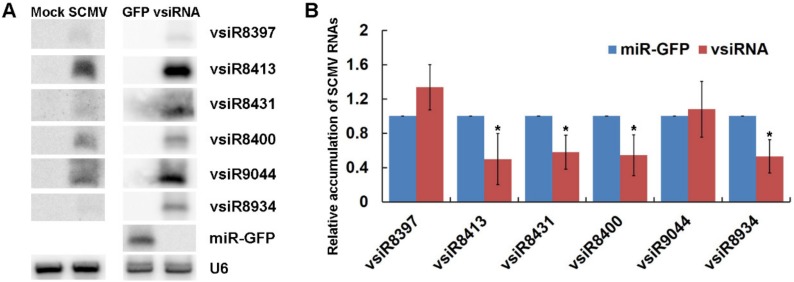
The expressed vsiRNAs could silence SCMV RNAs in maize protoplasts. (**A**) The accumulation of vsiRNAs was determined by Northern blotting in SCMV-inoculated maize plants and in maize protoplasts co-expressed with SCMV RNAs. Mock and SCMV indicated Mock- and SCMV-inoculated maize plants, respectively. GFP and vsiRNA indicated the maize protoplasts expressed miR-GFP and vsiRNAs, respectively. U6 was used as a loading control. (**B**) The relative accumulation of SCMV RNAs in maize protoplasts was determined by qRT-PCR when co-expressed miR-GFP or vsiRNAs. Three independent experiments were conducted with at least three biological replicates and the data were analyzed using a two-sample *t*-test. Bars represent the grand means ± SD. “*” indicate a significant difference (*p*-value < 0.05).

To investigate the roles of vsiRNAs in silencing SCMV RNA, amiRNAs were constructed to express six vsiRNAs with SCMV RNA in maize protoplasts, which possessed higher reads of correlated cleavage sites on SCMV genome and different nucleotides at the 5′-end (Table S6). Protoplasts co-transfected with pGD-amiR_528_-GFP and SCMV RNA were used as a control. The expressions of six vsiRNAs in maize protoplasts were determined by Northern blotting, whilst the accumulations of six vsiRNAs were detected in SCMV-infected maize plants (Figure 6A). The results showed that the amounts of vsiR8413 and vsiR9044 were higher than that of the other four vsiRNAs in both maize protoplasts transfected with vsiRNA-expressed plasmids and SCMV-infected maize plants (Figure 6A). The qRT-PCR analyses were then conducted on RNA extracts to quantify the relative expression levels of SCMV RNA. The results revealed that the expressions of vsiR8413, vsiR8431, vsiR8400 and vsiR8934 significantly reduced the accumulation of SCMV RNA (Figure 6B). Interestingly, the expressions of vsiR8397 and vsiR9044 with U at 5′-terminal that were preferentially loaded into AGO1 had no obvious effects on the accumulation of SCMV RNA, suggesting that AGO1 might not make a significant contribution to vsiRNA function in cleavage of SCMV RNA.

## 4. Discussion

In plants, RNA silencing is a conserved surveillance mechanism in the defense against viral infection, which can trigger the production of vsiRNAs in virus-infected plant cells [1]. Like miRNA, vsiRNAs were thought to silence target genes mainly by cleavage in plants [13]. As we know, decreased photosynthesis is typical in virus-infected plants. Moreover, our previous report has demonstrated that SCMV infection decreased the accumulation of photosynthesis-associated proteins and photosynthetic activity [36]. Two research groups simultaneously demonstrated that the bright yellow mosaic symptoms caused by *Cucumber mosaic virus* Y-satellite RNA are a result of siRNA-directed RNA silencing of the chlorophyll biosynthetic gene, providing direct evidence that the identified siRNAs derived from viral satellite RNA directly modulate the viral disease symptom by RNA silencing-based regulation of a host gene [37,38]. In this study, the degradome analysis was established to identify maize transcripts targeted by vsiRNAs from SCMV (Table S4). Forty-two different maize transcripts were identified as potential targets of vsiRNAs, six (*GRMZM2G009443_T01*, *GRMZM2G032628_T01*, *GRMZM5G806622_T01*, *GRMZM2G066234_T01*, *GRMZM2G130121_T01* and *GRMZM2G132847_T01*) of which might be involved in chloroplast development and function [33]. The qRT-PCR results revealed that SCMV infection down-regulated the accumulation of *GRMZM2G032628_T01* (Figure 2), a gene involved in starch biosynthesis [39] (Blauth et al., 2002). This study provided a new insight that chloroplast-associated genes might be regulated by vsiRNAs from SCMV, which resulted in the decreased photosynthesis and mosaic symptom; however, the molecular mechanisms remain to be investigated.

It has been revealed that vsiRNAs produced in the host cell can be incorporated into AGO-containing RISC to silence viral genome [1]. However, only a few vsiRNAs were demonstrated to be involved in the antiviral silencing [13,40]. The previous report showed that the majority of the vsiRNAs derived from *Tomato bushy stunt virus* (TBSV) were inefficient in guiding the formed RISC and only a distinct number rather than a broad variety of cleavage products were obtained, revealing that only some specific vsiRNAs might be highly effective [40]. In another report, only a few cleavage sites were identified in the viral genomes by degradome analysis, while a consistent fraction of vsiRNAs did not apparently account for cleavage, suggesting that only a low percentage of vsiRNAs were involved in the antiviral response [13]. In our study, large amounts of cleavage sites probably produced by vsiRNAs were identified in the sense strand of SCMV genomes (Figure 3 and Table S5), suggesting that most vsiRNAs from antisense strands of SCMV could effectively target SCMV genomic RNAs. However, only four cleavage sites existed in the antisense strand (Figure 3 and Table S5), indicating that abundant vsiRNAs from the sense strand of SCMV were inactive. Viral replication is associated with the virus-induced intracellular membranous structures that have been suggested to provide a scaffold for tethering the viral replication complex (VRC) to prevent the activation of certain host antiviral mechanisms, such as RNA silencing triggered by dsRNA intermediates during viral replication [35]. It was speculated that the antisense strand of SCMV as the template of replication could be protected by VRC from cleavage of vsiRNAs. These results further demonstrated that approximately equal ratios of sense and antisense SCMV vsiRNAs originated predominantly from dsRNA precursors produced by RNA-dependent RNA polymerase that processed the cleavage products of sense strand RNAs produced by antisense vsiRNAs [26].

It has been reported that amiRNAs highly accessible to targets confer efficient virus resistance in several plant species [18,19,20]. Transient expression of amiRNAs in protoplasts is a fast, easy and powerful tool for screening and assaying amiRNA activity without transgenic plants [16,21,22]. In this study, amiRNAs were successfully expressed in maize protoplasts based on the precursor of osa-miR528a (Figure 4C). However, the precursor of ath-miR319a as the backbone of amiRNAs was incompletely processed, which resulted in the low efficiency of amiRNA expression (Figure 4A,B). In a previous report, ath-miR319a-derived amiR-GFP-4 had high activity in maize protoplast at 36 h post transfection; however, the results of amiR-GFP-4 expression were not shown in Northern blotting assay [16], suggesting that the amiRNAs from the incompletely processed precursor might have activities enough to silence targets, or the time of incubation for protoplast post transfection in our study was too short. Anyhow, despite a certain level of cross-species activity in the expression and processing of plant amiRNAs derived from an exogenous miRNA backbone, it is perhaps most desirable to use the amiRNA backbone derived from the same or closely related plants to achieve optimal gene silencing in a given plant species [16].

RNA silencing as a plant immune system against viruses is triggered by the production of vsiRNAs that efficiently inhibit viral accumulation [1]. However, which vsiRNA functions efficiently silence the viral genome has been poorly studied. In this study, we established a transient expression system in maize protoplasts to investigate the effect of vsiRNAs on SCMV accumulation. The data showed that vsiR8397 and vsiR9044 had dramatically reduced or no activity (Figure 6), although they had U at 5′-terminal and relative accumulation, suggesting that AGO1 might play no or little role in vsiRNA function, or that the function of AGO1 was limited in maize protoplasts. Interestingly, the other four vsiRNAs could efficiently inhibit the accumulation of SCMV RNA (Figure 6B), indicating that other AGOs were recruited to form vsiRNA-RISCs and involved in anti-SCMV defense. The results of Northern blotting assays revealed that the accumulation of vsiRNAs had similar patterns in both transfected maize protoplasts and SCMV-infected maize plants (Figure 6A), further demonstrating the efficiency of a screen for vsiRNAs in transient expression system. The discrepant accumulation of different vsiRNAs under similar circumstances indicated that there was a strong correlation between the sequence and the accumulation of vsiRNAs; however, the detailed mechanisms need to be further investigated.

## 5. Conclusions

Virus infection triggers the production of vsiRNAs that can silence viral RNAs and specific host transcripts. However, which and how vsiRNAs function is poorly studied. Our previous studies have preliminarily characterized the vsiRNAs derived from SCMV and identified the target genes of maize miRNAs through degradome analysis. In this study, a degradome library was constructed and analyzed to identify the cleavage sites in viral and host transcripts mainly produced by vsiRNAs. We identified 42 maize transcripts that were possibly targeted by vsiRNAs, amang which several were involved in chloroplast functions and in biotic and abiotic stresses. Moreover, we obtained more than 3000 possible cleavage sites in positive-strand RNAs of SCMV, while there were only four cleavage sites in negative-strand RNAs. amiRNAs were designed and expressed in maize protoplasts to explore the roles of vsiRNAs in targeting SCMV RNAs. Four of the six vsiRNAs expressed by amiRNA technology could efficiently inhibit the accumulation of SCMV RNAs, suggesting that the transient expression system could effectively assess amiRNA activity of silencing viral RNAs *in vivo*. Further work will be needed to confirm the roles of vsiRNAs in targeting viral and host transcripts.

## Supplementary Materials

The following are available online at http://www.mdpi.com/1999-4915/10/12/664/s1, Table S1: The constructs and primers used in this study, Table S2: The probes for Northern blotting, Table S3: The primers used for qRT-PCR, Table S4: The targets of vsiRNAs identified from the degradome library of SCMV-infected maize leaves, Table S5: The cleavage sites on viral genomes targeted by vsiRNAs, Table S6: The information of vsiRNAs expressed in maize protoplasts.
